# Static Memory Deduplication for Performance Optimization in Cloud Computing

**DOI:** 10.3390/s17050968

**Published:** 2017-04-27

**Authors:** Gangyong Jia, Guangjie Han, Hao Wang, Xuan Yang

**Affiliations:** 1 Department of Computer Science and Technology, Hangzhou Dianzi University, No. 1108, Street 1, Xiasha, Hangzhou 310018, China; gangyong@hdu.edu.cn; 2 Department of Information and Communication Systems, Hohai University, Jinling North Road, No. 200, Changzhou 213022, China; wanghaohhu@outlook.com (H.W.); yangxuanhhu@outlook.com (X.Y.)

**Keywords:** main memory, memory deduplication, cloud computing, virtualization, performance

## Abstract

In a cloud computing environment, the number of virtual machines (VMs) on a single physical server and the number of applications running on each VM are continuously growing. This has led to an enormous increase in the demand of memory capacity and subsequent increase in the energy consumption in the cloud. Lack of enough memory has become a major bottleneck for scalability and performance of virtualization interfaces in cloud computing. To address this problem, memory deduplication techniques which reduce memory demand through page sharing are being adopted. However, such techniques suffer from overheads in terms of number of online comparisons required for the memory deduplication. In this paper, we propose a static memory deduplication (SMD) technique which can reduce memory capacity requirement and provide performance optimization in cloud computing. The main innovation of SMD is that the process of page detection is performed offline, thus potentially reducing the performance cost, especially in terms of response time. In SMD, page comparisons are restricted to the code segment, which has the highest shared content. Our experimental results show that SMD efficiently reduces memory capacity requirement and improves performance. We demonstrate that, compared to other approaches, the cost in terms of the response time is negligible.

## 1. Introduction

Cloud computing has become a popular computational technique in both industry and academia for reducing the cost of ownership and management of computational hardware while increasing the flexibility and on demand scalability of such resources [1]. Companies such as Netflix, Foursquare, and Snapchat [2], which serve millions of customers, have given up building their own computing infrastructure and moved their operations to cloud platforms such as Amazon Elastic Computing Cloud (EC2) [3] to easily scale their services. Everyday, more and more customers and enterprises are moving into cloud computing due to its low cost and high scalability [4,5,6].

Virtualization technology allows multiple operating systems to share computational resources by running multiple virtual machines (VMs) on a single physical server [7,8,9,10,11]. Since each VM can service a different client, several users can share the same physical resource thus decreasing the cost for all users. In order to decrease operating costs, companies providing virtualization technology try to accommodate as many clients as possible on a single physical server (current limits are up to eight VMs on a physical core in desktop cloud environments). However, increasing the number of VMs on a physical server, also leads to an increase in the memory requirements of the server. This increase in requirements of the memory capacity suffer from two bottlenecks: (a) increase in physical memory development is slower than the increase in memory demands; and (b) the increase in the energy consumption is very high for servers with more than 64 GB of memory [12,13,14]. This has turned memory capacity as one of the biggest challenges in cloud computing.

To alleviate memory demands, memory management techniques such as memory deduplication—which use page sharing of the same content into one single copy—have been developed. Both VMware and Difference Engine have reported that memory deduplication can help save memory across VMs. While VMware [15,16,17,18] states that about 40% memory can be saved, Difference Engine [19,20,21] reported that this number can go up to 50%. Due to its reported success, several systems have implemented memory deduplication techniques into their architecture. Linux implements Kernel Samepage Merging (KSM) [22], which is one implementation of memory deduplication. KSM is a Content-Based Page Sharing (CBPS) method, which adopts page granularity to detect duplicate content. There are two global red-black comparison trees in the KSM—one is a stable tree, and the other is an unstable tree. All shared pages are stored in the stable tree with write-protect policy, while all independent pages are stored in the unstable tree. Every candidate page is compared with pages in both stable and unstable tree and the trees are reordered accordingly.

One major drawback of using KSM is that it is part of the hypervisor (or Virtual Machine Monitor, VMM) and is implemented as a kernel thread that is periodically woken up for scanning. This periodic scanning can increase the response time of the system—especially when the scanning process runs along the critical path-leading to a sharp decrease in the system performance. Another major drawback of KSM is that it performs several unnecessary comparisons (of unshared content) which further increases the system load.

In this paper, we propose a static memory deduplication (SMD) approach aimed at optimizing the response time and reducing the number of unnecessary comparisons. In the SMD approach, we first partition the data into different zones according to the segment information of the VMs. Next, we analyze and compare only the zones which have a high probability of having duplicate contents. This decreases the overhead of multiple comparisons and leads to negligible drop in system performance [23,24].

In summary, in this paper, we provide the following contributions:

*Reduction in the number of comparisons*: based on a detailed profiling of the possible zones of duplicate content, we identify the code segment as having the highest probability to contain shared content. This allows us to restrict comparisons to the code segment and thus reduces the number of necessary comparisons and increases performance.

*Offline detection of same content pages*: We performed a detailed analysis of the overhead profiling of the KSM. Too many online comparisons increase the system response time, which is a critical performance metric in the cloud computing. In order to reduce this overhead, we propose the static memory deduplication (SMD) technique, which detects the same content pages offline.

*Limiting Page comparison to a single classification*: to reduce the overhead of performing unnecessary comparisons, without decreasing the opportunities of detecting same content pages, we first classify the pages into different categories based on their vector content. All pages of the same vector content are classified into a single category. Next, pages are only compared to those in their own category. This decreases the number of comparisons.

The rest of this paper is organized as follows. In Section 2, we elaborate the research motivations. In Section 3, we explain our static memory deduplication technique. In Section 4, we describe the experimental methodology and the results. Finally provide our conclusions in Section 5.

## 2. Motivation

### 2.1. Profiling Different Sharing Probability of Segments

**Figure 1:**
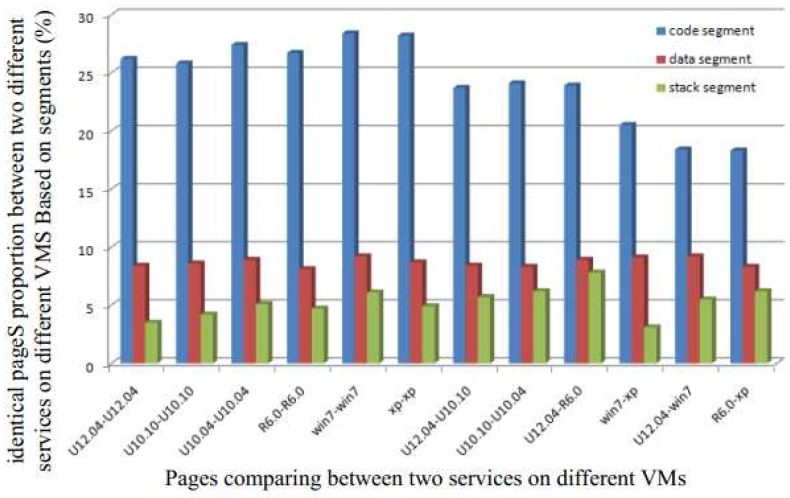
Proportion of identical pages for two different services running on different Virtual Machines for the different segments (code segment, data segment, and stack segment).

To decrease the possibility of having identical pages in different zones, we first partition the pages into three segments: (a) code segment; (b) data segment and (c) stack segment. Next, we analyze the proportion of identical pages in each segment. Figure 1 shows the proportion of identical pages between two different services running on two different VMs based on different segments. We observe that the highest possibility of having identical pages is in the code segment. This is easy to understand since pages in both the data and stack segments change frequently.

Therefore, even though there may be several identical pages in the system that can be shared through a single copy to reduce the memory requirement, the different segments offer different levels of efficiency. Detecting identical pages in the code segment is more efficient due to the static nature of this segment. It allows the detection to be performed offline. Although data and stack segments do have identical pages, their proportion is low, and hence the cost of duplicate detection increases. Moreover, the page contents of these two segments change frequently, and the overhead from frequent merging is not worth the gain obtained from removing duplicates.

Therefore, in this paper, in order to increase the efficiency of the system, we only focus on the code segment for detecting shared pages. This allows us to alleviate the memory capacity requirement and reduce the detection overhead. Moreover, since this detection can be processed offline, it reduces the load and hence the effect of the detection on the response time of system.

### 2.2. Comparison Overhead Analysis of KSM

KSM is the implementation of CBPS in the Linux kernel. It uses the scanning method and runs as a periodically scheduled kernel thread. In KSM, all pages of the system are partitioned into two groups. Each group is organized into two. There should be two trees, not one global red-black trees: one is called the *stable tree* and the other is called the *unstable tree*. All shared pages are in the stable tree, and all pages which are candidates for merging are in the unstable tree.

In each running period of the KSM, a candidate page will be compared with all pages in the stable tree. If a match in the stable tree is found, the candidate page will be deleted and the discovered content page in the stable tree will be used. Next, the page table entries will be replaced and then permission of the shared page will be changed to read-only. If a process attempts to write to the shared page, a Copy-On-Write (COW) fault will be triggered and the system will have to make a private copy for writing. If no match in the stable tree is found, the comparison will be performed on the unstable tree. If any identical page is found in the unstable tree, the candidate page will be merged and shared by replacing the page table entries and setting the permission of the shared page as read-only. Next, this page will be deleted from the unstable tree and be inserted into the stable tree. Similarly, if any attempt to write to the shared page occurs, a Copy-On-Write (COW) fault will be triggered as before. In case no duplicate page is found in either the stable or unstable tress, the candidate page will be inserted into the unstable tree.

KSM is not a scalable method—the duplicate detection cost is proportional to memory capacity. Therefore, in order to decrease the overhead of the scan speed, we need to carefully choose the mode of the scan. Although a fast scan can be more effective in detecting identical pages for short-lived page-sharing, it needs more CPU resources. This increases the response time for the scheduling threads of KSM. On the other hand, a slow scan can reduce CPU overhead and optimize response time. However, it is not effective in finding identical pages, especially in the case of short-lived page-sharing.

In KSM, candidate pages are partitioned into batches according to the number of pages. In a scheduled period of KSM thread, comparisons are only made within a single batch and then the KSM thread goes to sleep. Therefore, the scan speed can be optimized by optimizing both the size of the batch (the number of pages in a batch) and the sleep time between batches.

Table 1 and Table 2 show the different configurations used to perform the quantitative analysis of KSM in this paper. The experimental system has 4 GB of memory. The size of batch and the sleep time are the two metrics used to control the scan speed. They determine the sharing efficiency and run-time overhead. The configurations conf0 to conf4 are used to analyze the effect of the batch size for a constant sleep time of 30 ms, and the conf5 to conf9 are used to analyze the effect of sleep time for a constant batch size of 400.

**Figure 2:**
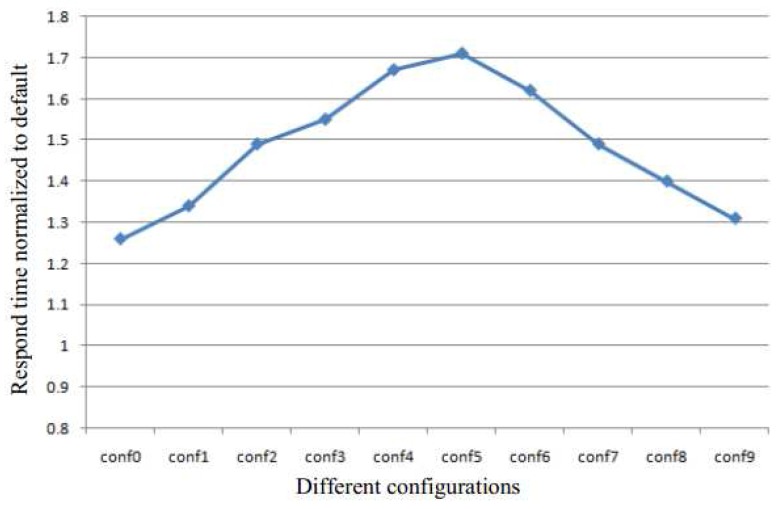
The response time normalized to default from conf0 to conf9.

Figure 2 demonstrates the response time normalized in the absence of KSM for configurations conf0 to conf9. As the size of the batch is increased, the response time is prolonged, as the sleep time is decreased. Therefore, in both sets of configurations, the performance of the system degrades.

**Figure 3:**
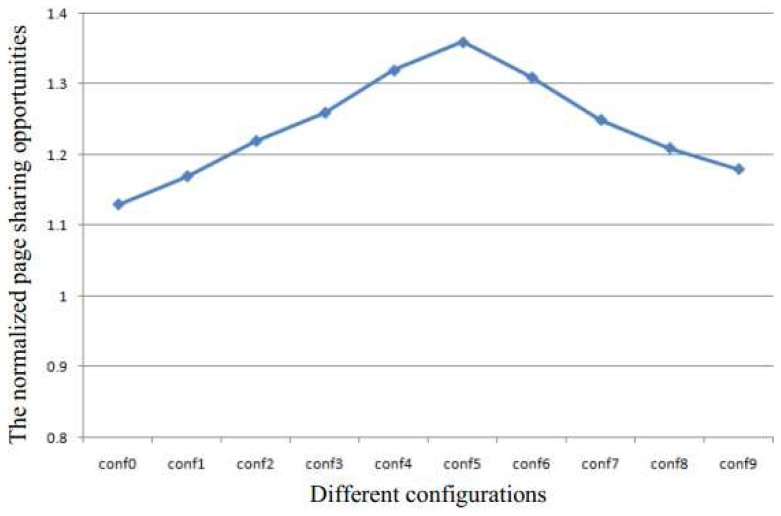
The normalized page sharing opportunities.

Figure 3 shows the normalized page sharing opportunities. The page sharing opportunities are increased in either case when the page sharing are increased or when the sleep time is decreased. Therefore, in both cases, the memory capacity requirement will decrease to improve system performance.

**Figure 4:**
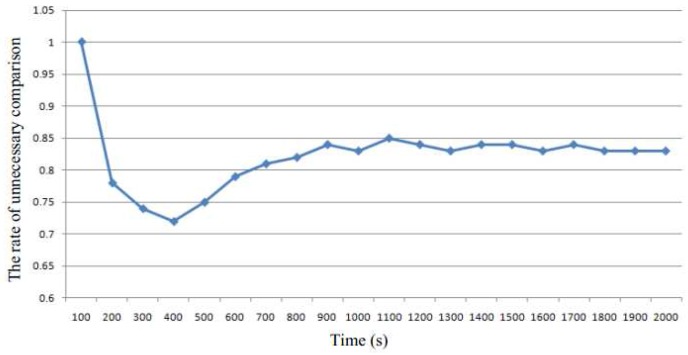
The rate of the unnecessary comparisons.

Figure 4 demonstrates the rate of unnecessary comparisons, which is one of the major overhead. The rate of unnecessary comparisons is defined as the number of unnecessary comparisons divided by the total number of comparisons. We see that the rate of unnecessary comparisons becomes steady at about 0.8, which means that most of the pages comparisons are unnecessary and increase the cost.

In summary, although the online memory deduplication technique can help detect identical pages, using KSM prolongs the response time of the system. Since reponse time is one of the major performance metrics of cloud computing, there is a need to decrease this. Moreover, there is a need to decrease the number of unnecessary comparisons to perform faster memory deduplication.

Therefore, the goal of this paper is to reduce the effect of the response time due to memory deduplication, while at the same time reduce the unnecessary page comparisons to save the processing required.

## 3. Static Memory Deduplication (SMD)

In Section 3.1, we provide an overview of the static memory deduplication (SMD) technique proposed by us. In Section 3.2, we discuss how to statically classify pages into the different categories, and, finally, in Section 3.3, we propose a lightweight memory deduplication approach to reduce unnecessary page comparisons.

### 3.1. Overview of SMD

In this section, we propose the static memory deduplication (SMD) technique to reduce the memory capacity requirement while keeping the response time same. It consists of two steps: (1) to reduce both the unnecessary page comparisons and the overhead of the response time; in the first step, we partition all pages into different categories according to the sampled content. We perform this offline to save processing power. Pages in the same categories have the same sampled content. The number of categories in the system is as large as the total number of content samples. The duplicate detection is restricted to each category, decreasing the CPU overhead; and (2) in the second step, we detect the identical pages within each category. If there is a page identical to the candidate page, then the candidate page is marked as a shared page and is linked to the other shared pages. In this way, all of the pages of the code segment are processed, and all shared pages are identified. After the process is scheduled, the shared pages can be merged to alleviate the requirement of memory and decrease the response time overhead.

**Figure 5:**
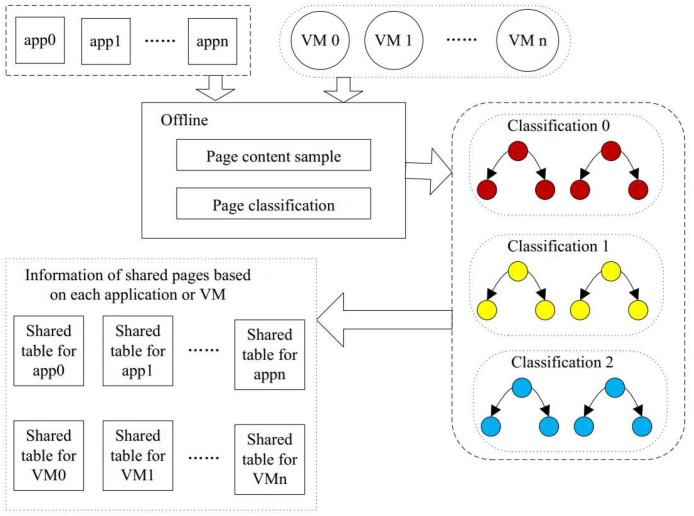
The Static Memory Deduplication (SMD) framework containing pages classification and each application or VM’s shared pages.

Figure 5 demonstrates our SMD approach, which contains the above two processes. The first process partitions all pages into categories according to the sample page content. Pages in the same category may be identical, but pages in the different categories will never be the same. The second process detects the identical pages within a category. We create a table of shared pages for each application or VM. The table of shared pages will contain all the pages of the application or VM which are shared with others. Moreover, the table will also provide the information of the other shared pages. Each application or VM has its own table of shared pages. Therefore, when running, the system can check this table to implement memory deduplication to reduce the memory capacity requirement.

### 3.2. Page Classification

The KSM approach is not efficient due to the large number of unnecessary page comparisons. In order to reduce the number of unnecessary comparisons, we classify all pages into different categories. The pages in the same category have higher chances of being shared, and the pages in the different categories are not shared. Therefore, the detection is restricted within a category. This page classification is implemented offline.

In order to do this, we follow the following steps. First, we sample each code page by sampling the page content. For example, we sample the content at fixed offset addresses, e.g., at the offset address 0, at the offset address 1024, at the offset address 2048, and so on.

Next, in the second step, we create a table, in which every entry is the head of a list. Furthermore, all of the pages of the same sampled result are stored in a single list. Therefore, all of the pages in a list are classified into a single category. In other words, the detection is restricted in one list. In case we increase the number of categories during the classification, then the number of comparisons decrease and the detection overhead becomes lower; however, the number of entries in the table increases.

In the third step, we insert the candidate page of application or VM into a list. According to the sampled result of the code page, if we find the same results of the entry in the table, we insert the page into this list.

Algorithm 1 demonstrates the above steps for page classification. The whole process is performed offline and is run on the code segment only. This is because pages from the other segments have less opportunities of being shared, and comparing all of them would decrease the effectiveness of the system by increasing the scanning cost.

**Algorithm 1** Page Classification algorithm (PCA)**Input:** 1:P: the sampled result of the candidate page 2:T: created table 3:$T_{i}$: the value of entry i in the table**Input:** 4:Partition application code or VM into pages; 5:Sample every page to get the result for each page; 6:**if** a sampled result is P **then** 7:  Find an entry in the table T, for which $T_{i}$ =P; 8:  Insert the page into the list i; 9:**end if**

### 3.3. Shared Pages Table for Each Application or VM

Since the KSM technique simply maintains two global comparison trees for all memory pages of a hosting server, to detect page sharing opportunities, each candidate page needs to be compared with a large number of uncorrelated pages in the global trees. Since this is performed online [7], it will increase the response time of system and slow it down.

In order to optimize the response time of system, we need to reduce the cost of this online detection with minimal effect on the sharing opportunities. Based on the page classifications, we propose a shared pages table for each application or VM. The shared table is used to record pages of the application or VM that have identical content with others. The shared page table is a variable size table, and, if the application or VM has many shared pages, the table is large; otherwise, the table is small. The table can even be null if the corresponding application or VM has no shared pages.

Each entry of the table demonstrates a list of identical content for each shared page. Thus, from the table, we can find all the identical pages in an application or a VM.

One important problem is how the shared pages table for each application or VM should be realized. In Algorithm 2, we demonstrate the process of implementation of the table of shared pages for every application or VM. First, we detect all the pages of the application or VM (restricting the detection to their corresponding page classifications). If a page has other the identical content pages, then one entry for the page is created in the shared pages table. Otherwise, we perform this detection for the next page. This process is performed offline, and hence does not affect the response time of system.

Through this shared pages table, the shared pages of the application or VM are all identified before being scheduled. Therefore, when the application or VM is running, all pages which can be merged have been marked, making the actual procedure of merging memory effective.

**Algorithm 2** The shared pages table implementation algorithm (SPTIA)**Input:**1:App: the application or VM2:$P_{i}$: the sampled result of the page i in the APP3:T: the shared pages table4:$T_{j}$: the entry j in the table**Output:**5:**while**
$P_{i}$
**do**6:  Find the page classification PC, for which $P_{i}$ ∈ PC;7:  Detect whether there are some pages with identical content;8:  **if** exists **then**9:    Create one entry in the shared pages table $T_{j}$;10:    List all pages having content identical to the $T_{j}$;11:    Continue;12:  **else**13:    Detect the next page Pi, which $P_{i}$ ∈ APP;14:    Continue;15:  **end if**16:**end while**

### 3.4. Implementation of Memory Deduplication

After the shared pages tables have been created for all the applications or VMs, the last step is to perform the actual memory deduplication. This step is performed online to alleviate the memory capacity request. Algorithm 3 shows the pseudocode of our implementation. First, we check all the pages of the running application or VM. For each page, if the page is in the shared pages table, it means that the page can be merged. Then, we find the pages that have identical content from the list in the shared pages table. If found, we revise the entry of the page table by making the entry of this page shared with the other pages with identical content. If not, then we check the next page of the running application or VM.

**Algorithm 3** Implementation of memory deduplication**Input:**1:App: the application or VM is running2:$P_{i}$: the page i in the APP3:T: the shared pages table4:$T_{j}$: the entry j in the table**Output:**5:**while**
$P_{i}$
**do**6:  Check whether $P_{i}$ is in T;7:  **if** yes **then**8:    Find the entry $T_{j}$ of the page in the table; 9:    Find the pages in the list $T_{j}$;10:    Revise the entry of $P_{i}$ in the page table of the App;11:    Continue;12:  **else**13:    Continue;14:  **end if**15:**end while**

## 4. Experimental Setup and Results

### 4.1. Experimental Setup

Table 3 shows the experimental configurations and Table 4 shows the workloads.

### 4.2. System Performance of SMD

We use the system throughput as the metric to define the system performance. System throughput is measured as the weighted speedup (as shown in Equation (1)). The $IPC_{i}$ represents the Instructions Per Cycle (IPC) of $VM_{i}$. Moreover, ${IPC}_{i}^{shared}$ denotes the Instructions Per Cycle (IPC) of $VM_{i}$ when running parellel with other VMs, and ${IPC}_{i}^{alone}$ denotes the Instructions Per Cycle (IPC) of $VM_{i}$ when running alone in the system:

(1) $$weighted\_speedup=\sum_{i}\frac{{IPC}_{i}^{shared}}{{IPC}_{i}^{alone}}.$$

**Figure 6:**
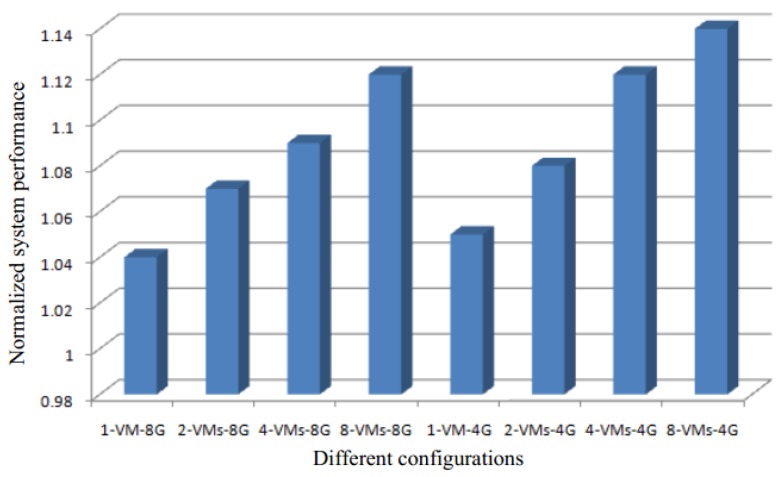
The normalized performance improvement for different configurations.

Figure 6 shows the normalized performance improvement for different configurations (from one virtual machine to eight virtual machines) with our SMD with with 4 G and 8 G memory and no memory deduplication. The *x*-axis represents the different configurations, with the different combinations of the parallelly running VMs number and total physical memory size. The n-VMs-mG represents that n VMs are running in parallel on a server with m G memory. Each VM has a different operating system.

The results of the figure show that increase in the number of VMs for the same amount of memory leads to a better system performance with our SMD approach. This is because, when a bigger number of VMs run in parallel on low memory, the number of memory requests increase. This configuration is able to optimally utilize the advantage of our SMD approach, and hence the system performance increases.

**Figure 7:**
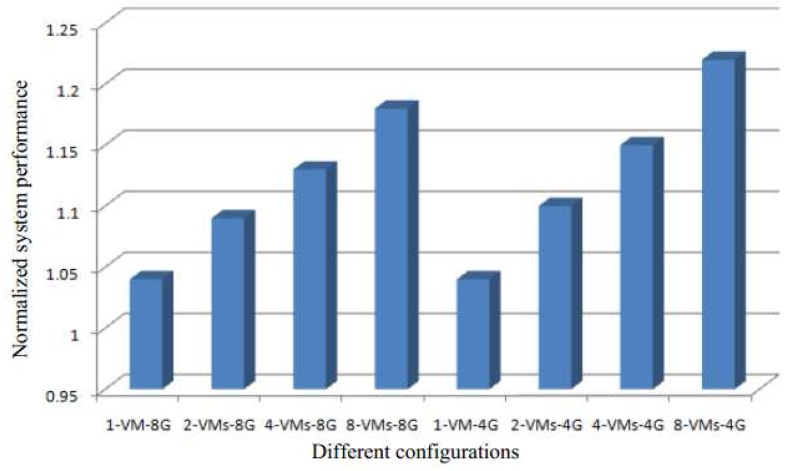
The normalized performance improvement when running same operating system for simultaneously VMs.

Figure 7 shows the normalized performance improvement when running the same operating system simultaneously on the VMs. The results show that running the same OS on the VMs lead to a better performance gain than when different operating systems are used. This is because the more identical the contents of the different systems are, the greater is the opportunity for identifying shared content. This can help to alleviate the memory capacity request and lead to better system performance.

**Figure 8:**
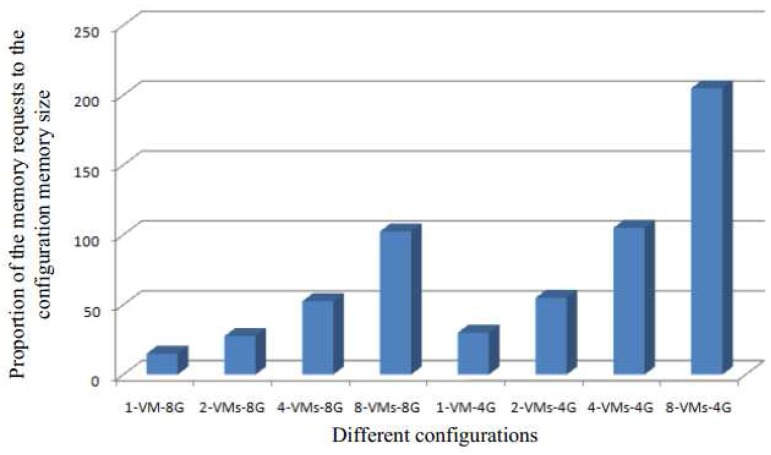
The proportion of memory requests for different configurations of the default system.

Figure 8 shows the proportion of memory requests for different configurations of the default system—from one VM to eight VMs with 4 G or 8 G memory. The memory contend is more serious if the proportion is high, which means that the more urgent it is to use memory deduplication to share the same content pages to reduce memory requests.

From Figure 7 and Figure 8, we can see the similar results, and their trends are almost the same. The results also prove that the larger the memory contend the greater opportunity for performance improvement.

**Figure 9:**
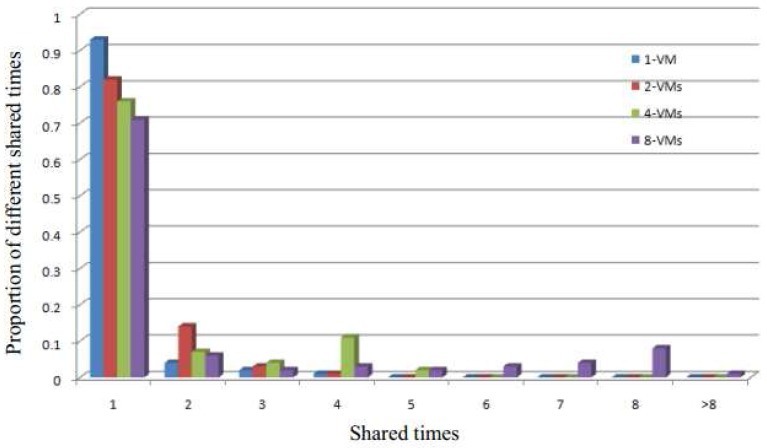
The proportion of physical pages for different shared times.

Figure 9 shows the proportion of shared times for the physical pages for different VMs. The results demonstrate that when there are some shared pages in the system, if the number of simultaneously running VMs increases, then there will be more sharing in the system. In addition, as the sharing in the system increases, so do the opportunities to merge the shared pages to alleviate the system memory capacity. Therefore, the results of the Figure 9 demonstrate that the greater the number of simultaneously running VMs, the better the performance of our SMD will be.

### 4.3. Response Time Optimization of SMD

The process of detecting shared pages using our SMD algorithm is performed is offline. This is different from the online detection approach used by the Linux KSM. The online detection of KSM affects the response time of system, degrading the system performance. Compared to KSM, our SMD has advantages in terms of better response time.

**Figure 10:**
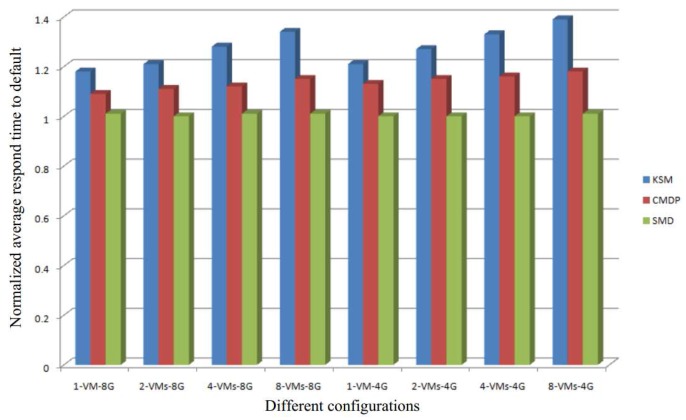
The normalized average response time of our system (SMD) compared to Kernel Samepage Merging (KSM).

Figure 10 shows the normalized average response time of our system compared to the response time of both KSM and CMDP [29]. The results show that our SMD has lower average response times under all configurations. Moreover, the more VMs running simultaneously in the system, the better response time of our SMD will be. The reasons for this can be summarized as follows:

(1) Unlike KSM and CMDP, our SMD has no online detection process. Since we perform the detection offline in our SMD, we obtain a great improvement in response time;
(2) In KSM and CMDP, an increase in the number of VMs simultaneously running in the system leads to an increase in the number of online comparisons. Therefore, the response time of KSM and CMDP decreases with an increase in the number of VMs. Since, in our SMD, the comparisons are performed offline, in our approach, the average response time is almost the same under all the configurations as demonstrated in Figure 10. This means that our SMD is highly scalable.
(3) The smaller the memory capacity of the system, the greater is the efficiency of the memory deduplication technique. For a system with small memory capacity, memory is the critical resource needed to improve the performance of the system. Thus, in the configuration of 4 GB memory capacity, the average response time of SMD is better than that of KSM and CMDP (see Figure 10).

**Figure 11:**
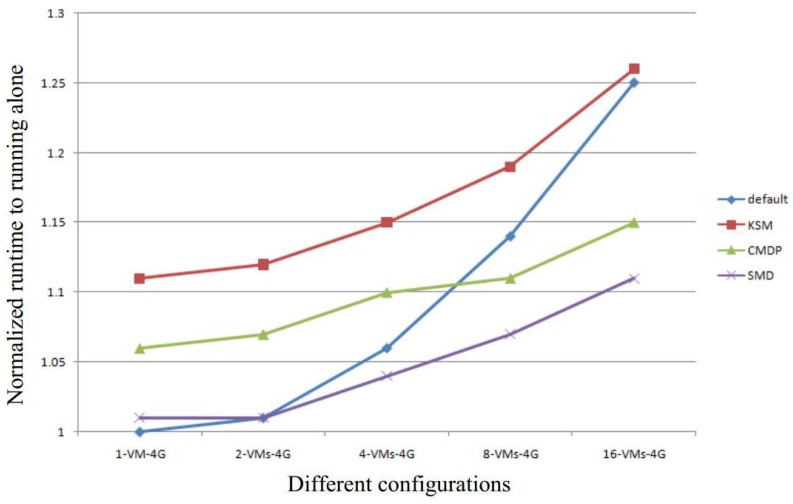
The average runtime of applications or VMs.

In both KSM and CMDP, the comparison is implemented as a kernel thread, and is scheduled periodically. When the thread is scheduled in the critical path, the performance of the system severely decreases. However, in our SMD approach, the comparison is static and hence will never be scheduled in the critical path. This allows for maintaining a higher performance of the system. Figure 11 shows the average completion runtime of applications or VMs. In the figure, we add the time of online comparisons to the runtime of applications or VMs. The results show that our SMD has the shortest runtime, which indicates that our SMD has the best performance. In the figure, the runtime of both KSM and CMDP are longer than default due to the time of comparison. However, when the number of VMs running simultaneously increases, the runtime of KSM is almost the same as the default. This shows that memory deduplication is good for improving system performance when more applications or VMs are run simultaneously.

### 4.4. Memory Capacity Request and Unnecessary Comparisons Reduction of SMD

**Figure 12:**
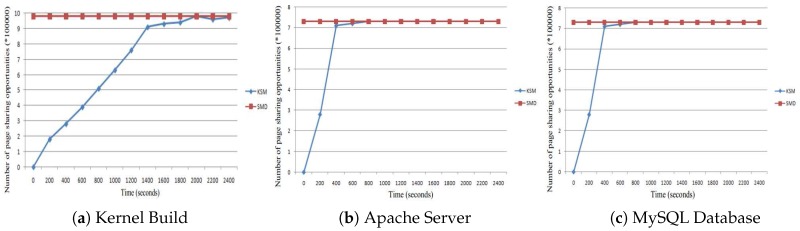
Page sharing opportunities with four VMs.

The page sharing opportunities are shown in Figure 12. The greatest page sharing opportunities that can be can be detected by KSM are shown in Figure 12a. However, to do this, a long time to reach the maximum opportunities is required. On the contrary, our SMD approach detects page sharing opportunities offline. Thus, even before the application or VM runs, the detecting process is finished. In the figure, we can see that from the very beginning, our SMD detects all of the sharing opportunities detected by the KSM during the run of the kernel build. Similar results are shown in Figure 12b,c for Apache and MySQL, respectively.

**Figure 13:**
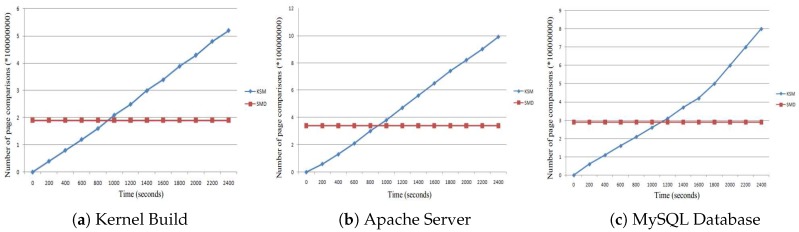
The number of page comparisons of different workloads with four VMs.

The number of page comparisons is shown in Figure 13. As shown in Figure 13, a KSM generates more comparisons for Kernel Build. SMD needs to perform only 32% of the total number of comparisons done by KSM. This is because our SMD technique restricts comparison of a page within a single classification. For Apache, the workload of page comparisons for SMD is 34% that of KSM. For MySQL, this number is further reduced to 33%.

**Figure 14:**
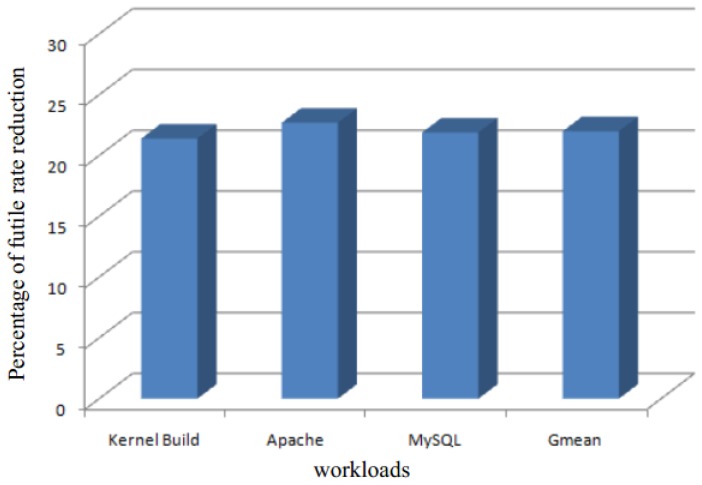
The percentage rate reduction of unnecessary comparisons with four VMs, where the baseline is from the KSM approach.

The percentage of unnecessary comparison rate reduction is shown in Figure 14. SMD, on average, can lead to a 22% decrease in the number of futile comparisons. Moreover, since SMD adopts page content samples, the reduction is almost the same for all workloads.

## 5. Conclusions

In this paper, we propose a static memory deduplication (SMD) technique to reduce memory capacity requirements for performance optimization in cloud computing. The SMD technique mainly contains three steps: (1) page classification, which classifies all pages into categories according to the content of the sampled pages; (2) in order to optimize the response time of the system, we need to reduce the number of comparisons during online detection without affecting the sharing opportunities. For this, we propose a shared pages table for each application or VM. The shared pages table for each application or VM is used to record pages of the application or VM that have identical content with others; (3) once the shared pages table for each application or VM has been built, we perform an online memory deduplication. This helps to alleviate the memory capacity requirement. The main innovation of our SMD technique is that we improve the performance by executing the process of page detection offline. Since through our experiments we identified that the code segment has the highest possibility of having duplicate content, in our SMD approach, the page comparisons are restricted to code segment. This leads to a decrease in the number of necessary comparisons and an increase in the performance. Our experimental results show that SMD can efficiently reduce memory capacity requirement and improve performance. Moreover, the page comparisons of SMD are negligible in terms of the system response time.

Although our proposed SMD approach is based on KVM in this paper, the SMD technique can be used for improving the performance of all other scan based memory deduplication techniques.

## Figures and Tables

**Table 1:** Different configurations of Kernel Samepage Merging (KSM).

Configuration	Conf0	Conf1	Conf2	Conf3	Conf4
size of the batch	100	200	400	800	1600
sleep time (ms)	30	30	30	30	30

**Table 2:** Different configurations of KSM.

Configuration	Conf5	Conf6	Conf7	Conf8	Conf9
size of the batch	400	400	400	400	400
sleep time (ms)	10	20	30	40	50

**Table 3:** System configurations for the experiment.

Parameters	Value
processor	Intel Xeon E5504 with EPT enabled, 2 × 4-core, hyper-thread disabled
L1 instruction/data cache	2-way, 32 KB
L2 cache	4-way, 256 KB
L3 cache	16-way, 4 MB
Hypervisor	QEMU [25] with KVM [26] (qemu-kvm-1.2.0), Ubuntu-12.04 with Linux
VM OS	kernel 3.6.0 64-bit Linux-10.10 with Linux kernel 2.6.32

KVM denotes Kernel-based Virtual Machine, QEMU denotes Quick Emulator.

**Table 4:** Workloads.

Parameters	Value
Kernel Build	compile the Linux kernel 3.6.10 in guest VMs
Apache Server	run the ab [27] benchmark on Apache httpd server with 24 concurrent requests
MySQL Database	run the SysBench [28] with MySQL database in guest VMs with 1-thread and
	configure the oltp-table-size as 1500000
